# Extraversion and neuroticism related to the resting-state effective connectivity of amygdala

**DOI:** 10.1038/srep35484

**Published:** 2016-10-21

**Authors:** Yajing Pang, Qian Cui, Yifeng Wang, Yuyan Chen, Xiaona Wang, Shaoqiang Han, Zhiqiang Zhang, Guangming Lu, Huafu Chen

**Affiliations:** 1 Center for Information in BioMedicine, Key laboratory for Neuroinformation of Ministry of Education, School of Life Science and Technology, University of Electronic Science and Technology of China, Chengdu, China; 2 School of Political Science and Public Administration, University of Electronic Science and Technology of China, Chengdu, China; 3 Department of Medical Imaging, Jinling Hospital, Nanjing University School of Medicine, Nanjing, China

## Abstract

The amygdala plays a key role in emotion processing. Its functional connectivity with other brain regions has been extensively demonstrated to be associated with extraversion and neuroticism. However, how the amygdala affects other regions and is affected by others within these connectivity patterns associated with extraversion and neuroticism remains unclear. To address this issue, we investigated the effective connectivity of the amygdala using Granger causality analysis on the resting-state functional magnetic resonance imaging data of 70 participants. Results showed that extraversion was positively correlated with the influence from the right inferior occipital gyrus (IOG) to the left amygdala, and from the bilateral IOG to the right amygdala; such result may represent the neural correlates of social interactions in extraverts. Conversely, neuroticism was associated with an increased influence from right amygdala to right middle frontal gyrus and a decreased influence from right precuneus to right amygdala. This influence might affect the modulations of cognitive regulation function and self-referential processes in neurotic individuals. These findings highlight the importance of the causal influences of amygdala in explaining the individual differences in extraversion and neuroticism, and offer further insights into the specific neural networks underlying personality.

Personality describes the integrated pattern of thoughts, feelings and behaviors that varies among individuals but remains stable within each individual across time^1^. Eysenck’s biological approach is perhaps the most influential model of human personality, suggesting that individual variations in emotional arousal can be described along three dimensions: extraversion, neuroticism and psychoticism^2^. Among these traits, extraversion and neuroticism are of particular importance in individuals because their brain correlates may contribute to a predisposition toward socio-emotional functioning and psychopathology^3^,^4^. Differences in these dimensions are known to influence emotional and cognitive processing.

Extraversion is linked to the tendency to experience positive emotions and social engagement^5^, which is typically stem from the tendency to respond positively to socio-emotional stimuli^6^. Conversely, neuroticism is related to increased susceptibility to negative emotion^7^, which is considered to originate from self-generated thoughts^8^. Moreover, individuals with high levels of neuroticism frequently experience difficulties in emotion regulation^9^ and demonstrate a negative self-referential information processing style^10^.

As a region critical to the organization of a complex series of emotional and physiological responses^11^, the amygdala has been centrally implicated in extraversion and neuroticism^12^,^13^. The afferents to and efferents from the amygdala have extensive connections with cortical and subcortical regions, and these connections allow them to participate in critical functions relevant to extraversion and neuroticism. These functions include the modulation of sensory information, such as the delivery of “gut feelings” about things that are good and bad [through the integration of afferents from all senses (e.g., visual, auditory, and somatosensory) as well as visceral inputs]^14^, emotion motivation and responses (through projections to cortical, hypothalamic and brainstem regions)^15^,^16^, and emotion regulation (through reciprocal connections to prefrontal and insular areas)^13^,^17^,^18^. Given the importance of the amygdala in emotion processing and its implication in extraversion and neuroticism, the information flow of the amygdala is expected to be fundamental to stable individual differences in these two key dimensions.

Correlation-based functional connectivity (FC) studies have revealed that functional coupling between the amygdala and distinct brain regions plays a crucial role in the individual differences in extraversion and neuroticism. Neuroticism was associated with amygdala-prefrontal [i.e., anterior cingulate cortex (ACC), ventromedial prefrontal cortex, and dorsolateral prefrontal cortex (dlPFC)]^19^,^20^,^21^, amygdala-hippocampus^21^, and amygdala-visual cortex connectivity^20^ during threat-related stimuli, possibly reflecting a general proneness to anxiety-related disorders in highly neurotic participants^20^,^21^. Extraversion was related to the neural pathways of the amygdala with ACC, insula, and thalamus regions in response to reward-related stimuli^13^. Resting-state functional connectivity (RSFC) studies also revealed that neuroticism is related to connections between the amygdala and brain regions involved in the processing of self-referential cues and emotion regulation [i.e., precuneus (PCu), prefrontal cortex (PFC), and visual cortex], whereas extraversion is related to the connections between the amygdala and brain regions implicated in socio-emotional function (i.e., insula, and occipital cortex)^12^,^22^. Individual differences in these amygdala connectivity patterns subserving cognitive-emotional functions are likely to be associated specifically with extraversion and neuroticism. A particularly interesting question is raised: What role does the amygdala play? To date, the manner in which the amygdala affects other regions and is affected by others within these connectivity patterns associated with extraversion and neuroticism has been unclear. The current study aimed to evaluate whether extraversion and neuroticism are associated with the causal influences between the amygdala and other brain regions at rest in a large sample of healthy subjects.

Effective connectivity (EC) referes to the influence that one brain region exerts over another^23^ and is evaluated through Granger causality analysis (GCA). This technique has been widely used for time-directed prediction among different blood oxygen level-dependent functional magnetic resonance imaging (fMRI) time series, and has revealed the pattern of causal influence among resting-state networks^24^,^25^, and among brain regions within the cortical-subcortical circuit related to affective disorders^26^,^27^,^28^. Thereafter, multiple regression analysis was implemented to determine the resting-state EC patterns of the amygdala which are associated with extraversion, and neuroticism. On the basis of neuroimaging literature, we hypothesized that extraversion would predict the process that the visual cortex affects the amygdala, which may partly explain the described bias towards social engagement^29^,^30^. Neuroticism would predict the interactions between the amygdala and the PFC and PCu regions, which may modulate the cognitive-emotional process in neurotic individuals^31^,^32^.

## Results

### EC of the amygdala is associated with extraversion

**Figure 1:**
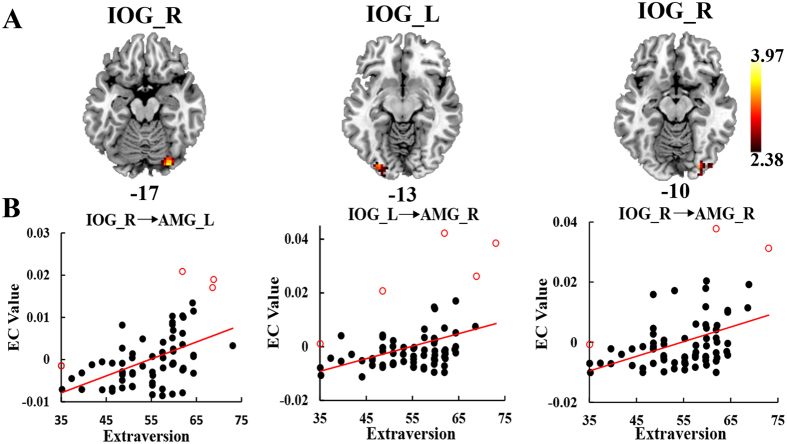
Regions of EC showing correlation with extraversion. (**A**) Extraversion was positively associated with the EC from the right IOG to the left AMG, the EC from the left IOG to the right AMG, and the EC from the right IOG to the right AMG. The hot color indicates the EC of the amygdala that show positive associated with extraversion. The color scale represents *t* values. (**B**) The scatter plots represent individual mean EC estimates from extraversion after regressing age, gender, FD, and neuroticism scores. The red circles are the outliers detected by bootstrapping the Mahalanobis distance. EC, effective connectivity; AMG, amygdala; IOG, inferior occipital gyrus.

Extraversion was positively correlated with the EC from the right inferior occipital gyrus (IOG) to the left amygdala, and from the bilateral IOG to the right amygdala (*p* < 0.05, AlphaSim corrected, Fig. 1, Table 1). Extraversion showed no significant correlation with the EC from the left and right amygdala to other brain regions.

### EC of the amygdala is associated with neuroticism

**Figure 2:**
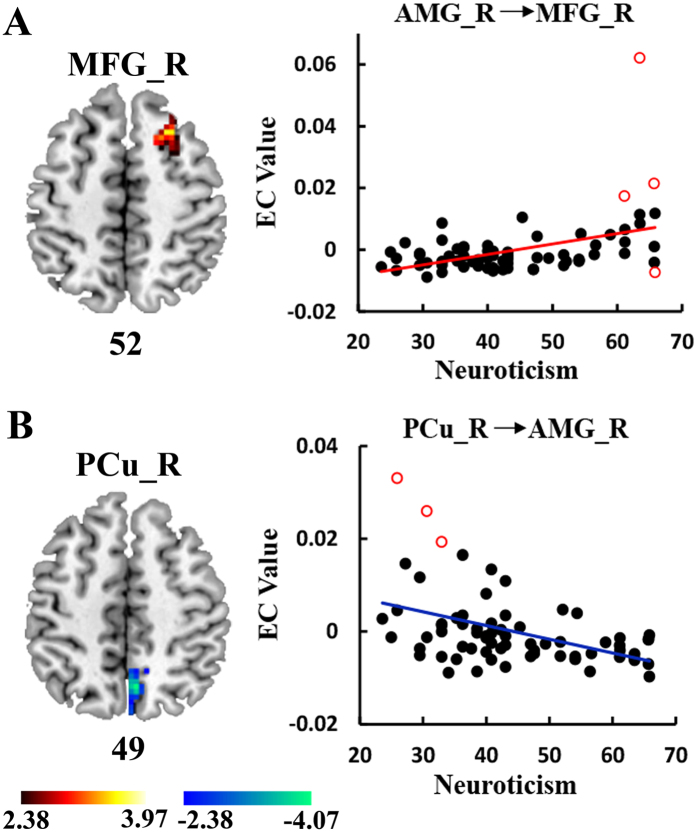
Regions of EC showing correlation with neuroticism. (**A**) Positive association between neuroticism and the EC from the right AMG to the right MFG. (**B**) Negative association between neuroticism and the EC from the right PCu to the right AMG. The hot and cold colors indicate the EC of amygdala that show positive and negative associated with neuroticism, respectively. The color scale represents *t* values. Right scatter plots represent individual mean EC estimates from neuroticism after regressing age, gender, FD, and extraversion scores. The red circles are the outliers detected by bootstrapping the Mahalanobis distance. EC, effective connectivity; AMG, amygdala; MFG, middle frontal gyrus; PCu, precuneus.

A positive correlation was observed between neuroticism and the EC from the right amygdala to the right middle frontal gyrus (MFG). Conversely, the EC from the right PCu to the right amygdala was negatively correlated with neuroticism (*p* < 0.05, AlphaSim corrected; Fig. 2, Table 2). Neuroticism showed no significant correlation with the EC of the left amygdala.

## Discussion

This study is the first to examine the relationship between extraversion, neuroticism, and the resting-state EC of the amygdala. Extraversion was found to be associated with an increased influence from visual processing streams to the amygdala. Neuroticism was associated with an increased influence from the amygdala to the MFG and a decreased influence from the PCu to the amygdala. These results excluded the influence of the hippocampus region, age, and gender. Moreover, the average EC maps of the amygdala across the sample revealed that the amygdala was connected to the PFC, occipital cortex, default mode network, and limbic regions (see Supplementary Figure S1); this finding was consistent with those of previous studies. The present findings indicate that individual differences in extraversion and neuroticism are associated with potential causal interactions between the amygdala and other distinct regions related to cognitive-emotional functions at rest.

### EC of amygdala is associated with extraversion

Our analysis showed that extraversion was positively correlated with the influences from the right IOG to the left amygdala, and from the bilateral IOG to the right amygdala. Extraverts who spend more time on interpersonal interaction are deemed to be better at processing faces than introverts^30^. The occipital face area pertains to the “core face-processing system” that mediates the visual analysis of faces^22^,^33^. The amygdala also belongs to the “extended” system that acts in concert with the core system to extract the meaning of information gleaned from faces^34^. The heightened activation of these regions was related to extraversion in response to positive social stimuli, such as happy faces^3^,^35^,^36^. The amygdala-visual cortex FC was implicated in attentional set^37^ and face recognition^22^, with the functions specially associated with the extraversion trait^30^. The amygdala receives the perceptual information from visual regions in processing socio-emotional stimuli and is involved in extracting emotional meaning to make appropriate responses, such as social judgments^29^, and attentional biases toward positive stimuli^38^. In this study, the increased connectivity between visual regions and the amygdala in extraverts might provide further neural pathway evidence of the relation between face recognition ability and social communication. Extraverts who are thought to be better at detecting and decoding the meaning of social cues have a better ability to navigate the dynamics of social interactions in social contexts than introverts^11^.

### EC of amygdala is associated with neuroticism

In the current study, a positive correlation between neuroticism and the influence from the right amygdala to the right MFG (BA8, dlPFC) was found. The amygdala-prefrontal network was shown to be associated with individual differences in the cognitive-emotional process^31^,^32^,^39^. The dlPFC mainly suppresses or promotes emotional responses controlled by the amygdala in complex scenes^40^,^41^. Alternatively, the amygdala conveys the emotional signal to the dlPFC region and is involved in handling the challenges to the organism by using different cognitive strategies^32^. According to these findings, people can modulate negative emotional responses through different cognitive strategies (i.e., suppression and reappraisal); in turn, the negative emotion can affect cognition function to determine behavioral outcomes in social surroundings^42^,^43^. These cognitive-emotional processes are strongly related to inter-individual variability in neuroticism^44^, which is a trait geared toward the emotional and cognitive processing of negatively affective stimuli^8^. Recent functional and structural connectivity studies have confirmed that the amygdala-PFC connectivity subserving emotion regulation processes is related to neuroticism^19^,^20^,^21^,^45^,^46^. With the support of the abovementioned findings, we suggest that the increased influence from the amygdala to the dlPFC might represent the neurobiological counterpart of the suboptimal cognition function associated with the internal negative emotion processes and characteristic of individuals with high neuroticism scores.

A negative association was found between neuroticism and the influence from the right PCu to the right amygdala. The PCu is implicated in self-related mental representations during rest^47^,^48^,^49^, especially among individuals high in neuroticism^50^,^51^, who tend to be more socially dysfunctional^52^. As shown in literature, the connectivity between this region and amygdala is important for different aspects of self-processing and for the modulation of the physiological response to emotion, and it contributes to the “disproportionate emotional coloring of self-referential processing”^12^,^52^. The amygdala-PCu pathway in the context of self-referential processing contributes to neuroticism^12^. Previous FC studies have likewise revealed that a decreased amygdala-PCu connectivity pattern is associated with high anxiety symptoms^53^,^54^. Thus, the current finding complements this notion and shows that neuroticism is associated with a decreased coupling between the amygdala and the PCu probably involving negative emotional coloring bias during states of spontaneous thought. This finding may imply that high levels of neuroticism are related to suboptimal self-relevant information processing.

### Potential confounding factors

To test the potential influence of the hippocampus, we calculated the EC between the hippocampus and other brain regions (see Supplementary S1). The results showed that only the influence from the PCu to the hippocampus positively associated with neuroticism was overlapped with the influence from the PCu to the amygdala negatively associated with neuroticism (see Supplementary Table S1). Therefore, the EC value was regressed out from the PCu to the hippocampus, and the result of neuroticism associated with a decreased influence from the PCu to the amygdala still remained (see Supplementary S3). These findings confirm the reliability of our results. Moreover, the effects of age and gender were assessed. We found no correlation between age and extraversion and neuroticism or between age and EC value (see Supplementary Figure S2). Thereafter, a consistent correlation was found between males and females (see Supplementary Figure S3). These pieces of evidence confirm that our findings are not confounded by age and gender.

Many inferenced studies on extraversion and neuroticism used the NEO questionnaire is derived from the Costa and McCrae five-factor model (Extraversion, Neuroticism, Agreeableness, Conscientiousness, and Openness)^55^. This model and the Eysenck three-factor model are the most influential and widely used models in the field of personality structure^56^. Despite differences between the two models, the results of factor analysis suggested that extraversion and neuroticism were highly convergent with the corresponding trait measures in both systems^56^,^57^. The relationship between Psychoticism from the Eysenck model and Agreeableness, Conscientiousness, and Openness factors from the Costa and McCrae model had no clear conclusion^57^,^58^. Previous neuroscience studies have suggested that the neural mechanisms underlining extraversion and neuroticism across the two models were similar^59^,^60^,^61^. According to literature, these two systems measure the same or at least highly overlapped traits, namely, extraversion and neuroticism.

### Limitation

The interpretation of our causal influence between the amygdala and other brain regions is restricted by the inherent limitations of GCA measures. Although GCA is widely used to model the causal influence that one neural time series exerts on another, it has some drawbacks in fMRI applications. It should be noted that systematic differences across brain regions in hemodynamic lag can potentially lead to spurious estimations of causality^62^,^63^. Until now, arguments are still existing on whether this disturbance will affect the studies at the group level^64^,^65^,^66^. This factor is an important limitation in considering, for example, the argued influence of the amygdala over dlPFC. In addressing these issues, the analyses of data collected through short TRs may help. To some extent, our findings hold potential for the causal connectivity of the amygdala related to extraversion and neuroticism. Future neuroimaging studies based on specific tasks and some interventions are needed to test the precise associations between the amygdala and other regions underlining the individual differences in extraversion and neuroticism.

## Conclusion

In summary, we provide evidence on the association of individual differences in extraversion and neuroticism and the EC pattern of the amygdala. In particular, extraversion was positively correlated with the influence from the visual cortex to the amygdala, which may represent the neural correlates of social interactions in extraverts. Conversely, neuroticism was positively associated with the influence from the amygdala to the MFG and negatively correlated with the influence from the PCu to the amygdala. This finding may underline the mechanism of suboptimal cognitive regulation function and self-referential processes in neurotic individuals. These findings indicate that the causal interactions between the amygdala and other brain regions related to cognition-emotional function are associated with individual differences in extraversion and neuroticism. The implementation of the resting-state EC of the amygdala in this study may help advance our understanding of the complex neural mechanisms of personality.

## Methods

### Participants

Seventy young, healthy, right-handed volunteers (34 males; age range: 19–26 years, mean age: 22.3 years, SD = 1.5) participated in this study. The participants had no history of neurological disorders or head injury and were not receiving any medications. The study was approved by the local Medical Ethics Committee of Jinling Hospital, Nanjing University School of Medicine, and was carried out in accordance with the approved guidelines. All participants provided written informed consent before any study procedure was initiated.

### Personality questionnaires

Personality dimensions were assessed using the Eysenck Personality Questionnaire-Revised Short Scale for Chinese (EPQ-RSC)^4^,^67^. The EPQ-RSC is a self-reported questionnaire that includes four dimensions of extraversion (*E*), neuroticism (*N*), psychoticism (*P*), and lie (*L*), with each dimension measured by 12 true-or-false questions. Each dichotomous item was scored with 1 or 0, and each dimension had a maximum possible score of 12 and a minimum of 0. As two basic and significant dimensions, extraversion and neuroticism were used for measuring correlations with EC values in this study. In the present study, raw extraversion scores ranged from 3 to 12 (M = 8.97, SD = 2.24), and raw neuroticism scores ranged from 0 to 9 (M = 3.33, SD = 3.10). Then, the initial extraversion and neuroticism scores were converted into T scores through the formula^67^:


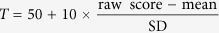


where mean represents the mean value of the personality scores over the normative sample; SD is the standard deviation of the personality scores over the normative sample. Subjects yielded mean values of M = 54.61 (SD = 8.44; score range: 35.07–73.05) on the extraversion dimension and M = 44.31 (SD = 12; score range: 23.57–65.82) on the neuroticism dimension. Furthermore, the distribution of extraversion scores was skewed negatively (skew = −0.51; kurtosis = −0.09), and the distribution of neuroticism scores was skewed positively (skew = 0.35; kurtosis = −0.92). A significantly negative correlation was found between extraversion and neuroticism through Pearson's correlation (*r* = −0.40, *p* < 0.0001).

### Image acquisition

Resting-state fMRI data were acquired using a 3T Siemens Trio scanner (Jinling Hospital, Nanjing, China). Foam pads and headphones were used to minimize head movement and scanner noise for all subjects. During data acquisition, the participants were instructed to simply rest with their eyes closed, relax their minds, not fall asleep, and not move if possible. Functional images were collected transversely using a gradient-recalled echo-planar imaging sequence with the following parameters: repetition time (TR)/echo time (TE) = 2000/30 ms, flip angle (FA) = 90°, field of view (FOV) = 24 cm, slices = 30, in-plane matrix = 64 × 64, and voxel size = 3.75 × 3.75 × 4.40 mm^3^. For each subject, a total of 250 volumes were acquired.

### Data preprocessing

Data preprocessing was performed using the SPM8 software (Statistical Parametric Mapping, http://www.fil.ion.ucl.ac.uk/spm/software/spm8). The first ten volumes were discarded due to the instability of the initial MRI signal and the adaptation of participants to the experimental procedure. The remaining 240 volumes were first corrected by the acquisition time delay among different slices and then realigned to correct for head-motion. No subject was excluded under the head motion criterion of ±1.5 mm and ±1.5°. Subsequently, the corrected images were spatially normalized to the Montreal Neurological Institute template and resampled to 3 × 3 × 3 mm^3^ cubic voxels. Filtering (0.01 Hz–0.08 Hz) and linear detrend removal were implemented to remove low-frequency drift and minimize high-frequency physiological noises^68^. As an additional motion correction, we calculated the frame-wise displacement (FD)^69^ to express instantaneous head motion, and the threshold of 0.5 was suggested. The maximum FD value was 0.33, and no subjects’ FD values were beyond 0.5. The M ± SD of FD over subjects was 0.12 ± 0.0630. The correlation analysis showed no significant correlation between extraversion and FD (*r* = −0.21, *p* = 0.09) and a positive correlation between neuroticism and FD (*r* = 0.24, *p* = 0.04).

### EC analysis

Recently, GCA and dynamic causal modeling (DCM) have become two state-of-the-art approaches for estimating EC from fMRI data^70^,^71^. DCM requires a prior specification of an anatomical network model and then tests the best possible networks^72^. By contrast, GCA only requires the predefined of regions of interest (ROIs) and can be applied directly to detect the coupling between ROI and other regions, without prior knowledge of the connections model between regions^73^. Accordingly, GCA was used with the left and right amygdala as ROIs to conduct the EC analysis. ROIs derived from the automated anatomical labeling template implemented in the MarsBaR toolbox (http://marsbar.sourceforge.net) were created. For each subject, the time series in the ROIs and the rest of the voxels of the whole brain were extracted. Several nuisance covariates including six rigid body head motion parameters, white matter, and cerebrospinal fluid signals, were regressed through linear regressions to remove the source of spurious variances^74^,^75^,^76^.

Residual-based voxel-wise GCA on the was performed through the in-house MATLAB toolbox (http://guorongwu.weebly.com/software.html)^77^,^78^ to describe the EC between the seed regions (left and right amygdala) and all other brain regions in each subject. In this study, the preprocessed mean time course of the seed region was defined as the time series x, and y denotes the time series of all other brain regions. The bivariate residual GCA was conducted to calculate the liner directional influence from x to y (*F*_*x*→*y*_) and from y to x (*F*_*y*→*x*_)^79^,^80^,^81^,^82^. Thus, two Granger causality maps (i.e., *F*_*x*→*y*_ and *F*_*y*→*x*_ maps) were generated for each subject and for each seed region. The order of the autoregressive model was set to 1 according to the Schwartz criterion. The coefficients of the models were calculated using a standard least squares optimization. Finally, the Granger causality maps were spatially smoothed by convolution with an isotropic Gaussian kernel (FWHM = 4 mm).

### Statistical analysis

A voxel-based multiple regression analysis (based on the general linear model) was implemented to map the effect of personality dimensions on amygdala EC, with EC value (i.e., the mean value of the left amygdala with *F*_*x*→*y*_ and *F*_*y*→*x*_ and the right amygdala with *F*_*x*→*y*_ and *F*_*y*→*x*_) as the dependent variable, and extraversion and neuroticism scores of the personality test as the covariates of interest. In addition, age, gender, and FD were used as external regressions to control their effects on the association between extraversion, neuroticism and brain EC^59^,^83^.

Given the theoretical and statistical anticorrelation between extraversion and neuroticism, the variance specific to each dimension should be partial out to obtain effects that were uniquely driven by each personality dimension. Therefore, we added extraversion (or neuroticism) scores as covariate of no interest in the model when calculating the relationship between neuroticism (or extraversion) and the EC of the amygdala. The correction for multiple comparisons used a combined height-extent threshold calculated through Alphasim Monte-Carlo simulation with 1,000 iterations. The corrected statistical threshold (*p* < 0.05) was accomplished as follows^84^. The regression analysis result was corrected (*p* < 0.05/8) [2 (extraversion/neuroticism) × 2 (left/right amygdala) × 2 (direction)] by the AlphaSim program in the REST 1.8 software (http://www.restfmri.net/forum/REST_V1.8), with each multiple regression analysis (with a voxel-wise threshold of uncorrected *p* < 0.01 with a minimum cluster size of 95 connected voxels for the relationship between extraversion and the left amygdala with *F*_*x*→*y*_, 98 voxels for the relationship between extraversion and the left amygdala with *F*_*y*→*x*_, 105 voxels for the relationship between extraversion and the right amygdala with *F*_*x*→*y*_, 126 voxels for the relationship between extraversion and the right amygdala with *F*_*y*→*x*_, 84 voxels for the relationship between neuroticism and the left amygdala with *F*_*x*→*y*_, 89 voxels for the relationship between neuroticism and the left amygdala with *F*_*y*→*x*_, 110 voxels for the relationship between neuroticism and the right amygdala with *F*_*x*→*y*_, and 100 voxels for the relationship between neuroticism and the right amygdala with *F*_*y*→*x*_).

## Additional Information

**How to cite this article**: Pang, Y. *et al*. Extraversion and neuroticism related to the resting-state effective connectivity of amygdala. *Sci. Rep.* **6**, 35484; doi: 10.1038/srep35484 (2016).

## Supplementary Material

Supplementary Information

## Figures and Tables

**Table 1:** The relationship between extraversion and the EC of the amygdala.

Brain Regions	Brodmann Area	MNI			Cluster size (voxels)	Peak *t*-value
		x	y	z		
other regions→amygdala_L						
IOG_R	18	33	−96	−12	103	4.34
other regions→amygdala_R						
IOG_L	18	−51	−63	−18	145	3.59
IOG_R	18	30	−87	−12	138	3.37

Regions show the EC of the amygdala. EC, effective connectivity; IOG, Inferior occipital gyrus; L, left; R, right. Positive and negative *t*-values indicate positive and negative correlations between extraversion and the EC of the amygdala, respectively.

**Table 2:** The relationship between neuroticism and the EC of the amygdala.

Brain Regions	Brodmann Area	MNI			Cluster size (voxels)	Peak *t*-value
		x	y	z		
amygdala_R→other regions						
MFG_R	8	30	9	66	139	3.91
other regions→amygdala_R						
PCu_R	7	6	−63	48	119	−4.07

Regions show the EC of the amygdala. EC, effective connectivity; MFG, Middle frontal gyrus; PCu, Precuneus; L, left; R, right. Positive and negative *t*-values indicate positive and negative correlations between neuroticism and the EC of the amygdala, respectively.
