# Supplementary material for: Extraversion and neuroticism related to the resting-state effective connectivity of amygdala
Source: Sci Rep. 2016 Oct 21;6:35484. doi: 10.1038/srep35484 (PMC5073227; doi:10.1038/srep35484)
Supplement: Supplementary Information [file srep35484-s1.pdf]

Extraversion and neuroticism related to the resting-state effective connectivity of amygdala

Yajing Pang[1#], Qian Cui[2#*], Yifeng Wang[1], Yuyan Chen[1], Xiaona Wang[1], Shaoqiang Han[1], Zhiqiang Zhang[3], Guangming Lu[3], Huafu Chen[1*]

[1] Center for Information in BioMedicine, Key laboratory for Neuroinformation of Ministry of Education, School of Life Science and Technology, University of Electronic Science and Technology of China, Chengdu, China

[2] School of Political Science and Public Administration, University of Electronic Science and Technology of China, Chengdu, China

[3] Department of Medical Imaging, Jinling Hospital, Nanjing University School of Medicine, Nanjing, China

[#]Yajing Pang and Qian Cui contribute equally to this work

Word count (exc. references, tables and figure legends): 3798

* Corresponding authors:

Huafu Chen: chenhf@uestc.edu.cn

Center for Information in BioMedicine, Key laboratory for Neuroinformation of Ministry of Education, School of Life Science and Technology, University of Electronic Science and Technology of China, Chengdu 610054, China

Or

Qian Cui: qianwang26@gmail.com

School of Political Science and Public Administration, University of Electronic Science and Technology of China, Chengdu, China

S1 EC analysis of the hippocampus

The amygdala is structurally located adjacent to the hippocampus and that strong interconnection between these regions has been shown in healthy adults[1]. In general, the signal of the amygdala might be polluted by the hippocampus. In order to comprehensive demonstrate the association between human personality and the resting-state effective connectivity (EC) of the amygdala, we performed a complementary EC analysis of the hippocampus to evaluate the influence on the results of amygdala. Thus, we conducted additional seed-based EC analyses of the hippocampus.

The left and right hippocampus regions of interest were also created by using the automated anatomical labeling template which implemented in the MarsBaR toolbox. The EC of the hippocampus was also calculated by using the residual-based voxel-wise GCM, the same as the calculation of the EC of amygdala. Finally, the obtained GC maps of the hippocampus were spatially smoothed by convolution with an isotropic Gaussian kernel (FWHM = 4 mm).

S2 Statistical analysis

A voxel-based multiple regression analysis was implemented to map the effect of personality dimensions on hippocampus EC. The procedures were the same as mapping the effect of personality dimensions on amygdala EC. The corrected statistical threshold (p < 0.05) was also accomplished the same as amygdala. In detail: the regression analysis result was corrected (p < 0.05/8) [2 (extraversion/neuroticism) x 2 (left/right amygdala) x 2 (direction)] by the AlphaSim program in the REST 1.8 software (http://www.restfmri.net/forum/REST_V1.8) with each multiple regression analysis (with a voxelwise threshold of uncorrected p < 0.01 with a minimum cluster size of 109 connected voxels for the relationship between extraversion and the left hippocampus with $F_{x \to y}$, 108 voxels for the relationship between extraversion and the left

hippocampus with $F_{y\rightarrow x}$, 79 voxels for the relationship between extraversion and the right hippocampus with $F_{x\rightarrow y}$, 83 voxels for the relationship between extraversion and the right hippocampus with $F_{y\rightarrow x}$, 91 voxels for the relationship between neuroticism and the left hippocampus with $F_{x\rightarrow y}$, 95 voxels for the relationship between neuroticism and the left hippocampus with $F_{y\rightarrow x}$, 78 voxels for the relationship between neuroticism and the right hippocampus with $F_{x\rightarrow y}$, and 82 voxels for the relationship between neuroticism and the right hippocampus with $F_{y\rightarrow x}$).

S3 EC of the hippocampus related to neuroticism

The results show that neuroticism was negatively correlated with the EC from the left hippocampus to left Culmen, while positively correlated with the EC from the left precuneus (PCu) to the right hippocampus ($p < 0.05$, AlphaSim corrected; Table S1). We found no significant association between extraversion and the EC of the hippocampus.

In order to ensure whether the EC of hippocampus and PCu influence the EC results of amygdala and PCu, we added the mean EC value of hippocampus and PCu as covariate of no interest in the model when calculating the relationship between neuroticism and the EC of amygdala and PCu. The result of neuroticism was negatively correlated with the EC from right PCu to right amygdala still remained.

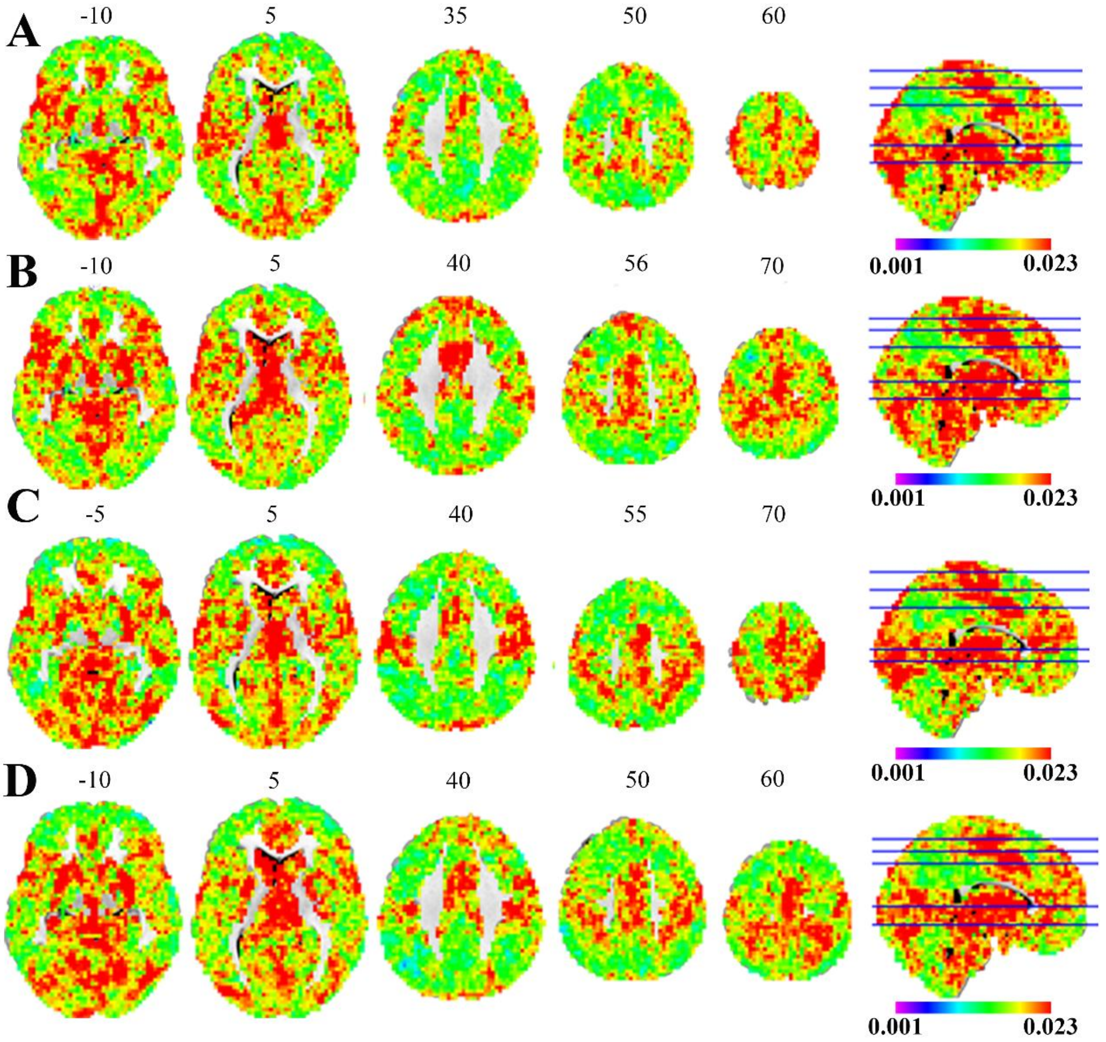

Supplementary Figure S1. (A)The mean EC value from left amygdala to other brain regions; (B)The mean EC value from other regions to left amygdala; (C) The mean EC value from right amygdala to other brain regions; (D) The mean EC value from other regions to right amygdala.

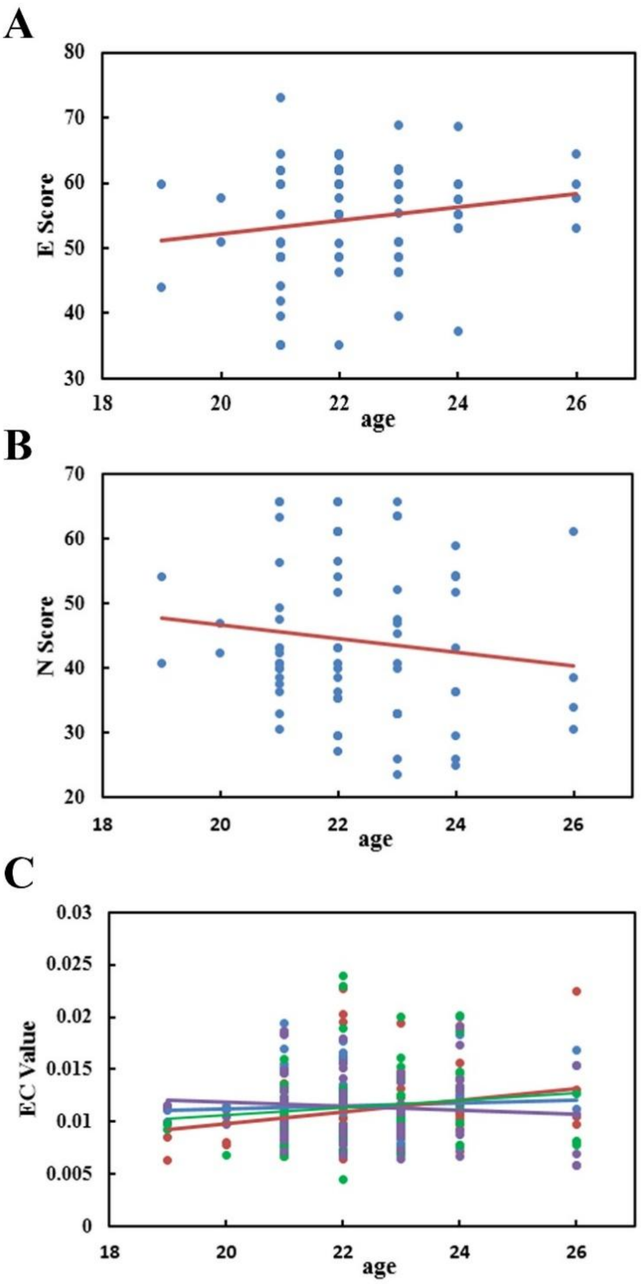

Supplementary Figure S2. (A) The association between age and extraversion (r = 0.18, p = 0.13), (B) between age and neuroticism (r = -0.13, p = 0.28); (C) between age and the mean EC value (r = -0.04, p = 0.75 for EC from the left amygdala to the whole brain in blue; r = -0.07, p = 0.55 for EC from the whole brain to the left amygdala in purple; r = 0.14, p = 0.25 for EC from the right amygdala to the whole brain in red; r = 0.03, p = 0.81 for EC from the whole brain to the right amygdala in green).

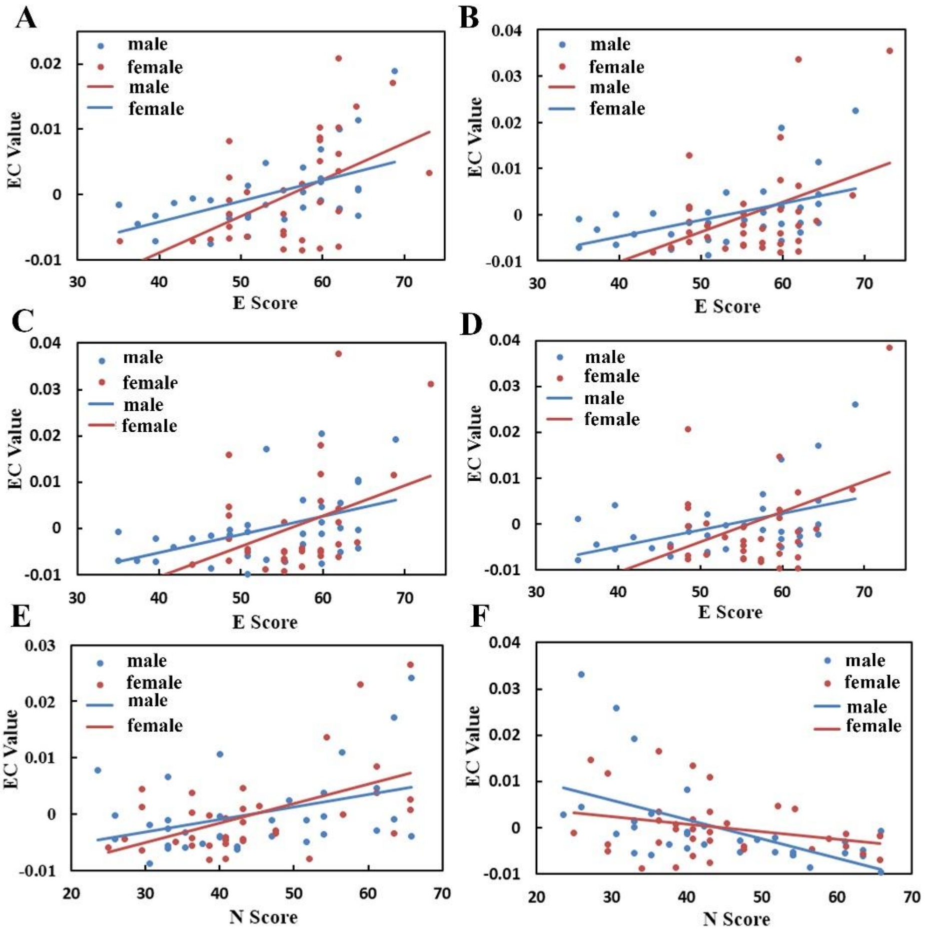

Supplementary Figure S3. (A) The correlation between extraversion and the EC value of the left amygdala and right IOG (r = 0.55, p = 0.007 for males; r = 0.35, p = 0.04 for females); (B) between extraversion and the EC value of the right amygdala and left FG (r = 0.51, p = 0.002 for males; r = 0.46, p = 0.005 for females); (C) between extraversion and the EC value of the right amygdala and right IOG (r = 0.48, p = 0.004 for males; r = 0.43, p = 0.007 for females); (D) between extraversion and the EC value of the right amygdala and left IOG (r = 0.47, p = 0.004 for males; r = 0.40, p = 0.01 for females); (E) between neuroticism and the EC value of the right amygdala and right MFG (r = 0.39, p = 0.02 for males; r = 0.52, p = 0.001 for females); (F) between neuroticism and the EC value of the right amygdala and right PCu (r = -0.57, p = 0.0004 for meals; r = -0.39, p = 0.04 for females). The results show that the tendency of correlation was consistent between males and females. There were no sex differences in EC.

Supplementary Table S1. The relationship between neuroticism and the EC of the hippocampus

**Brain Regions**	**Brodmann Area**	**MNI**			**Cluster size (voxels)**	**Peak *t*-value**
		x	y	z		
hippocampus_L→ other regions						
**Culmen_L**	19	-3	-54	-15	98	-3.71
other regions→hippocampus_R						
**PCu_L**	19	-33	-81	33	236	4.48

Regions shows the EC with the hippocampus. PCu, Precuneus; L, left; R, right. Positiveand negative *t*-value indicate positive and negative correlations between neuroticism and the EC of the hippocampus, respectively. No significant findings were observed for extraversion.

1 Roy, A. K. *et al.* Functional connectivity of the human amygdala using resting state fMRI. *NeuroImage* **45**, 614-626 (2009).
